# Experimental and Numerical Investigation of Intact, Defective and Repaired Countersunk Composite Joints under Tensile Loading

**DOI:** 10.3390/ma15134677

**Published:** 2022-07-04

**Authors:** Yongjie Huang, Zhidong Guan, Xian Yi, Zhangsong Ni, Tian Ouyang, Zengshan Li

**Affiliations:** 1 Chengdu Fluid Dynamics Innovation Center, Chengdu 610010, China; huangyongjie@buaa.edu.cn; 2 School of Aeronautic Science and Engineering, Beihang University, Beijing 100191, China; o.y.tian@buaa.edu.cn (T.O.); lizengshan_buaa@163.com (Z.L.); 3 China Aerodynamics Research and Development Center, Mianyang 621000, China

**Keywords:** countersunk composite joints, defects, repair, mechanical behaviors, finite element

## Abstract

Bolted joints are commonly used for assembling carbon fiber/resin composite structures. Since drilling may generate defects at hole edges which affect mechanical properties, it is of great engineering significance to develop proper repair methods to restore the mechanical properties of the defective parts. However, there are few studies on hole edge defects and their repair methods. Therefore, a novelty short fiber filling repair method was proposed to repair defective holes in this study. The mechanical properties of intact, defective and repaired countersunk composite joints were compared and investigated. Experimental tensile tests showed that defective joints had lower initial stiffness and failure loads compared to intact joints, while the mechanical properties were effectively restored after repair. Three-dimensional finite element models were also established to analyze the damage process of the joints. Results of numerical modelling were consistent with the experimental results. The simulations showed that changes in contact behaviors and local deformations caused by hole edge defects led to the low initial stiffness and stiffness transition point of the joint, while this phenomenon was reduced after repair. Additionally, despite different joint types, laminate failure mainly occurred around the hole and countersink.

## 1. Introduction

Composite materials have been used in the aviation industry for more than forty years as they provide the high specific stiffness/strength required for the challenging operating conditions. Three types of joints are commonly used in aircraft composite structures: bolted joints, bonded joints and hybrid bolted/bonded joints [1]. Among them, bolted joints are the most common type used for joining different composite parts, and many studies have been conducted regarding the mechanical behavior of such structures.

The hole clearance has a significant influence on the mechanical behavior of composite bolted joints. Mccarthy et al. [2] conducted a series of experiments to evaluate the effects of hole clearance on the stiffness and ultimate strength of single-lap composite joints. The results showed that increasing the clearance reduced joint stiffness and increased ultimate strains. Moreover, a delay in initial load take-up, which might change load distributions in multi-bolt structures, was also observed for joints with a large clearance value. Compared to composite joints without clearance, Scalea et al. [3] found that the compressive stresses in holes of composite joints with clearance were much higher due to the reduced contact area. Zhai et al. [4] performed single-lap, single-bolt composite joint bearing tests, showing that both the bolt-hole clearance and torque had an influence on the failure load.

In addition to experiments, advanced three-dimensional (3D) finite element (FE) simulations have also been widely used to analyse stress states and damage processes in composite structures [5,6,7]. Qin et al. [8] showed that different contact areas and local deformations between composite joints with protruding shear bolts and countersunk bolts caused different stress distributions near the hole edge. Stocchi et al. [9] established a very detailed FE model of composite joints with countersunk bolts to accurately determine the stress state and contact between the holes and bolts. Additionally, many studies have employed Hashin-type failure criteria and the constant degradation law to predict the failure loads of composite bolted joins; the numerical results agreed well with the experimental results [10,11,12,13,14]. Hashin-type failure criteria were shown to be computationally efficient in FE models.

Previous research has mainly focused on intact composite bolted joints with hole edge clearances. However, in the manufacturing process, especially during manual drilling operation, composite bolted joints may easily get damaged due to unreasonable operations or accidents, and have defects, such as over-tolerance holes, burrs, spalling and cracks, which should also be considered [15,16]. The effect of these defects on the joint may be greater than that of ordinary hole edge clearance. Some researchers have studied the relationship between drilling parameters and hole defects, and the results showed that drilling quality was a key factor affecting the mechanical properties and service life of composite joints [17,18,19]. Therefore, it is quite important to evaluate the residual strength and stiffness of defective joints in engineering.

In addition to the hole edge defect, the need of maintenance and repair for the defective holes is also a major concern for both the manufacturers and the end-users. In order to ensure the safety of structures in service, it is necessary to repair defective composite joints to avoid damage propagation from the defects and restore their strength and stiffness as much as possible. Current mainstream repair methods for composite structures include external patch bonding [20], scarf repair [21], bolted repair [22] and resin injection [23] techniques. However, most of these methods are used to repair penetration damage, impact damage or delamination defects and there are rare reports of repairing defective holes. Therefore, the repair method for defective holes and the mechanical properties of the repaired structure also need to be further studied, which is of great significance to the practical application of composite joints in aeronautical engineering.

In this article, in order to realize an economic and practical maintenance solution, a short fiber filling repair method (filling the geometric defects with short fiber composite materials and redrilling the hole into the intact size) was proposed to repair defective countersunk composite joints. Through quasi-static uniaxial bearing tests, the mechanical behaviors of intact, defective and repaired single-lap single-bolt composite joints were compared, and the effectiveness of the repair method was evaluated. Then, to better understand the tensile loading and damage process of the joints, 3D FE models considering progressive failure analysis were developed for each type of joint. Based on the simulation results, laminate/bolt contact behaviors and damage modes of different types of joints were discussed.

## 2. Materials and Methods

**Figure 1:**
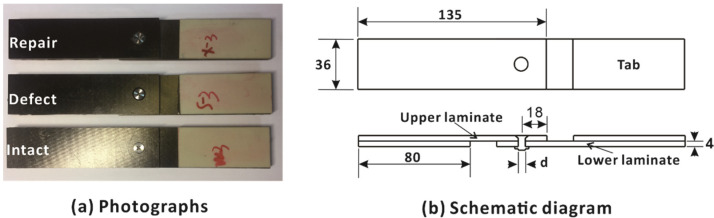
Photographs and schematic diagram of the tensile test specimen (all dimensions in mm).

As shown in Figure 1a, three types of single-lap single-bolt countersunk composite joints were analyzed in this study, which were intact, defective and repaired, respectively. For each type, there were 3 samples, and altogether 9 samples were used. All samples were manufactured from T700/QY9611 carbon fiber/bismaleimide composites with a lay-up of [45/90/−45/0/45/−45/90/0]_2s_ (widely used in aircraft structures). The joint samples and their basic geometry are shown in Figure 1b.

The intact joints were prepared according to the recommendations in ASTM D5961/ D5961M. The nominal ply thickness was 0.125 mm, and the total thickness of the laminate was 4 mm. The mechanical properties of the lamina, measured by basic mechanical experiments, are listed in Table 1.

**Figure 2:**
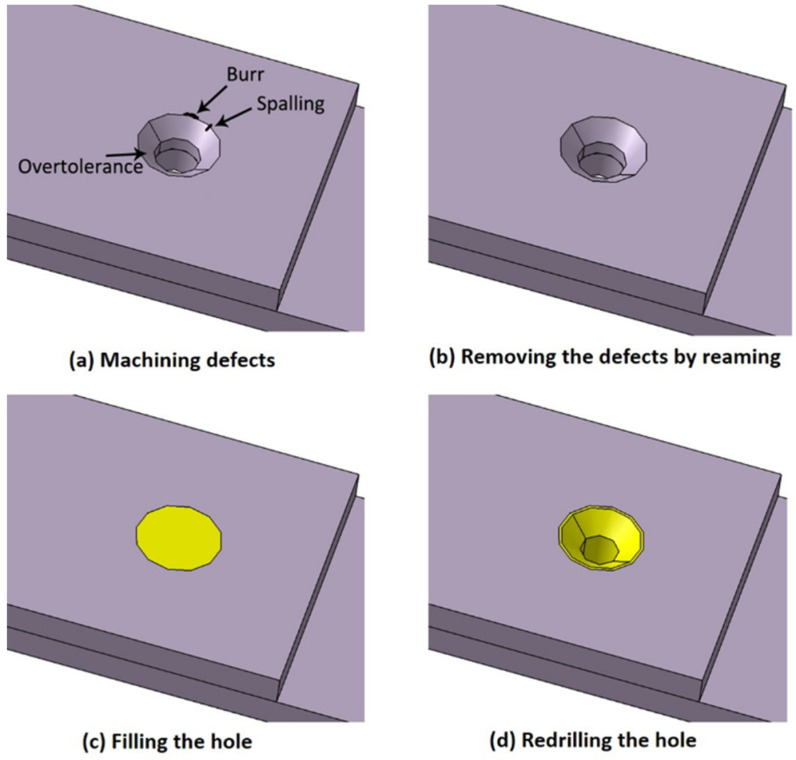
Illustration of the short fiber filling repair method for repairing defective composite joints.

As mentioned above, due to the quality of the manual drilling process, machining defects, such as over-tolerance, burrs and spalling may appear on the hole of a composite joint (see Figure 2a). In order to eliminate the impact of defects, the defective countersunk composite joints were repaired using a short fiber filling repair method. The repair procedure in this study involved three steps (see Figure 2b–d): removing the machining defects by reaming, filling the hole with short fiber composite materials, then redrilling the hole to the intact size.

**Figure 3:**
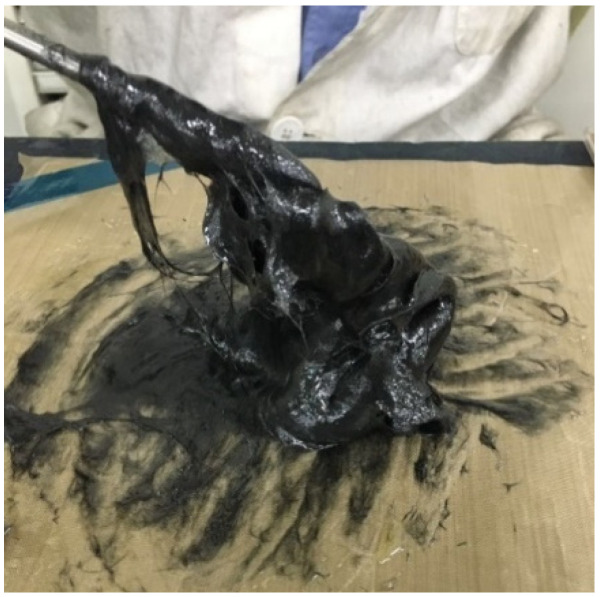
Short fiber composite materials before curing.

The short fiber composite material was prepared using a mixture of T700 short fibers and DG-3 epoxy adhesive (made in China), as shown in Figure 3.

The lengths of the short fibers were 2 to 3 mm, and the fiber fill fraction in the mixture was 3%. The fibers and DG-3 epoxy adhesive were well mixed to produce a mixture with randomly oriented fibers. Before filling the defective hole with the short fiber composite, the surface of the hole was cleaned with alcohol. The laminate samples with repaired holes were vacuumed to −0.8 MPa using a vacuum system and cured at room temperature (26 °C) for 48 h. After curing, the filler material had an elastic modulus of 10 GPa, Poisson’s ratio of 0.36, and compression strength of 290 MPa (measured by basic mechanical tests and shown in Table 2).

**Figure 4:**
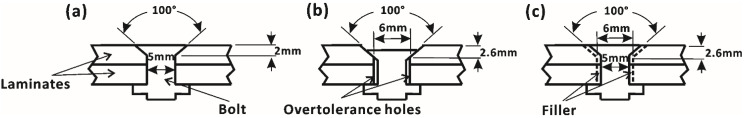
Geometric details of (**a**) intact, (**b**) defective and (**c**) repaired samples.

The detailed geometries of three sample types are shown in Figure 4. In this study, the defective samples had over-tolerance holes which represented the most severe case of geometric defects. The diameter of the holes in intact samples was 5 mm, while that of defective samples was 6 mm (with a larger size to represent geometric over-tolerance holes). The initial diameter of the holes in repaired samples was also 6 mm. Short fiber composite materials were used to fill the defective holes, and then the holes were drilled again to a diameter of 5 mm with a 100° countersink, nominally identical to the intact laminates; this process is shown in Figure 2. XH714 vertical machining centers and diamond-coated carbide drills were used for drilling. The spindle speed was 500 rpm, and the feed speed was 110 mm/min. Aerospace-grade titanium alloy bolts with a diameter of 5 mm and 100° countersink were used for all composite joint samples. A torque of 1 Nm was applied using a calibrated torque wrench.

**Figure 5:**
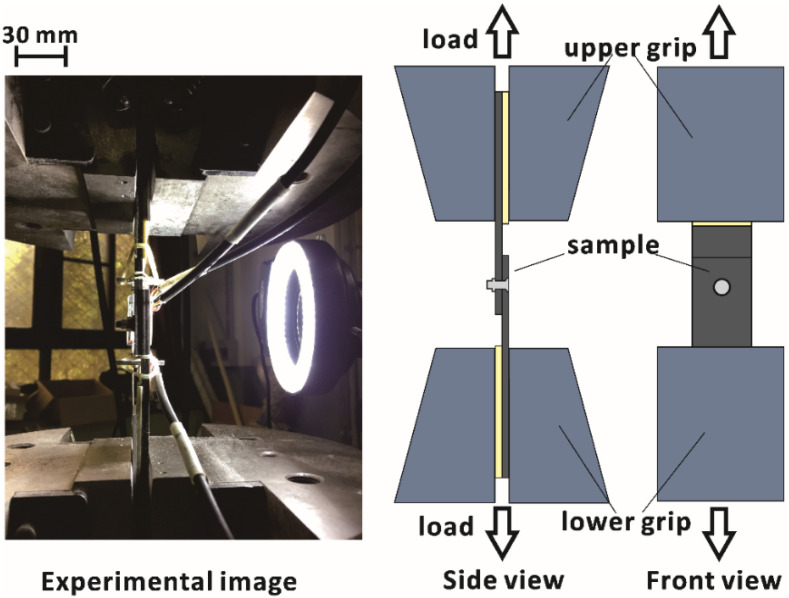
Schematic diagram of the tensile test.

All tensile tests were performed using an Instron 8803 machine with a load capacity of 250 kN at room temperature. The system was hydraulically driven, and the load was applied in displacement control mode at a rate of 2 mm/min. The applied load and grip holder displacement were automatically recorded by the system software. The sample clamping method used in the tests is shown in Figure 5. The tests were stopped when catastrophic failure of the samples occurred or the load suddenly dropped 30% from the peak value.

## 3. Experimental Results

### 3.1. Load-Displacement Curve

**Figure 6:**
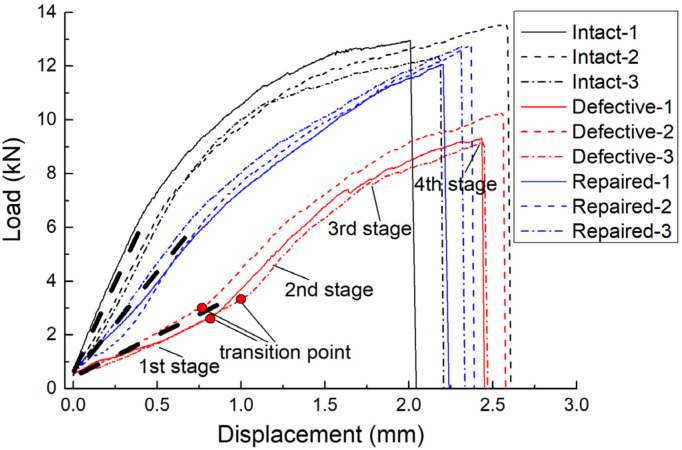
Load-displacement curves of intact, defective and repaired samples.

The load–displacement curves of the intact, defective and repaired samples are shown in Figure 6. It can be seen that curves of intact joints included three stages. In the first stage, the curves were almost linear before the loads reached about 6.5 kN. The slopes of the curves (stiffness of the joint) then gradually decreased during the second stage, as the bearing damage in the holes gradually expanded. Finally, the curves suddenly dropped when the countersunk bolts fractured due to shear stresses and the joints could no longer bear loads.

In the case of the repaired samples, the curves were similar to those of the intact samples, including a linear region of constant stiffness, a gradual decrease in stiffness and finally a sharp drop due to catastrophic shear-out damage. However, the stiffness of the repaired joints was lower than that of the intact joints.

In contrast to the intact and repaired curves, the load–displacement curves of the defective samples showed four distinct stages. In the first stage, the joint showed a low stiffness when the load was below 3 kN as the bolt shank could not completely bear the load. There was a clear transition in the stiffness between the first and second stages, where the stiffness in the second stage was higher than that in the first stage. In the third stage, with the increase of load, the propagation of bearing damage reduced the stiffness of the joint. Finally, the curve sharply dropped when the bolt fractured.

In general, due to the influence of hole edge geometric defects, the initial stiffness (and hence, bearing capacity) of defective samples was much lower compared with the other composite joints, especially at low load conditions. The repair method used here increased the stiffness of defective composite joints, although the original stiffness of the samples was not fully restored.

### 3.2. Failure Load

**Figure 7:**
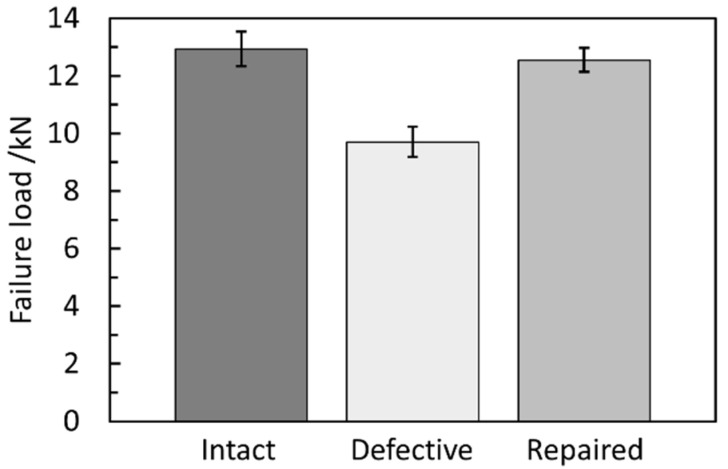
Failure loads of the intact, defective and repaired composite joints.

The failure loads of the intact, defective and repaired composite joints are shown in Table 3 and Figure 7. The variation coefficients of three types of joints are all within 6%, which reflects the validity of the experiment. The average failure load of the defective samples was 9.70 kN, while that of the intact samples was 12.94 kN. The average failure load of defective joints was only 74.9% of the intact joints; hence, defective holes greatly reduced the strength of the composite joint. The average failure load of repaired samples was 12.55 kN, which was 96.9% of the intact samples, indicating that the proposed repair method could compensate the geometric defects and almost restore the strength of the joint. Moreover, compared with the intact joints, the lower and upper limit of load capacity recovery ratio of the repaired joints were 93.4% and 99.1%, respectively, representing excellent repair efficiency.

### 3.3. Failure Mode

Although bolt shear-off was the final failure mode of intact, defective and repaired composite joints, obvious bearing damage was observed in the holes of three sample types before final failure. Therefore, the experimental data were effective.

**Figure 8:**
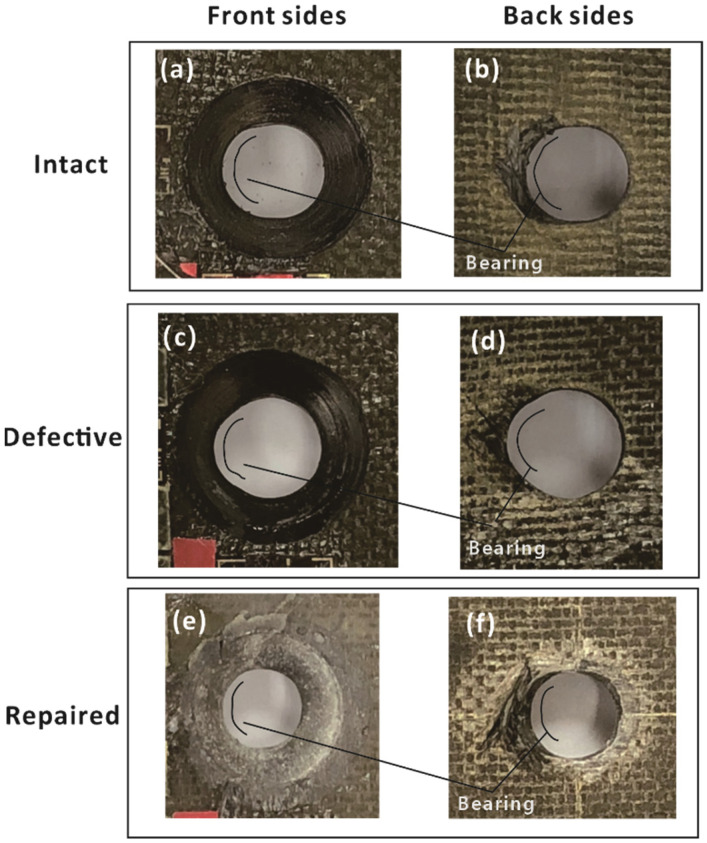
Failure modes of intact, defective and repaired samples. Front sides of (**a**) intact, (**c**) defective and (**e**) repaired samples; back sides of (**b**) intact, (**d**) defective and (**f**) repaired samples.

Figure 8 shows the failure modes of the upper laminates, where parts (a,b), (c,d) and (e,f) correspond to the intact, defective and repaired samples, respectively. Figure 8a,c,e show the front sides of the upper laminates, while Figure 8b,d,f show the back sides of the upper laminates. It can be seen from the figure that bearing damage was the main failure mode for all joints. Since the initial hole diameter of the defective joint (6 mm) was larger than that of the intact and repaired joints (5 mm), the contact area between the bolt and hole of the defective sample was less that of the other samples. Therefore, more pronounced bearing deformations occurred in defective samples compared to intact and repaired samples, as shown in Figure 8. This indicates that stress concentration may be one of the causes of the early fracture of the bolt in the defective joint, resulting in the lower failure load (see Table 3). In the case of the repaired samples, the shape and extent of hole deformation were similar to those of the intact samples. Moreover, Figure 8f shows that the filler material near the hole of the laminate was almost crushed.

## 4. Numerical Analysis

### 4.1. FE Modelling

**Figure 9:**
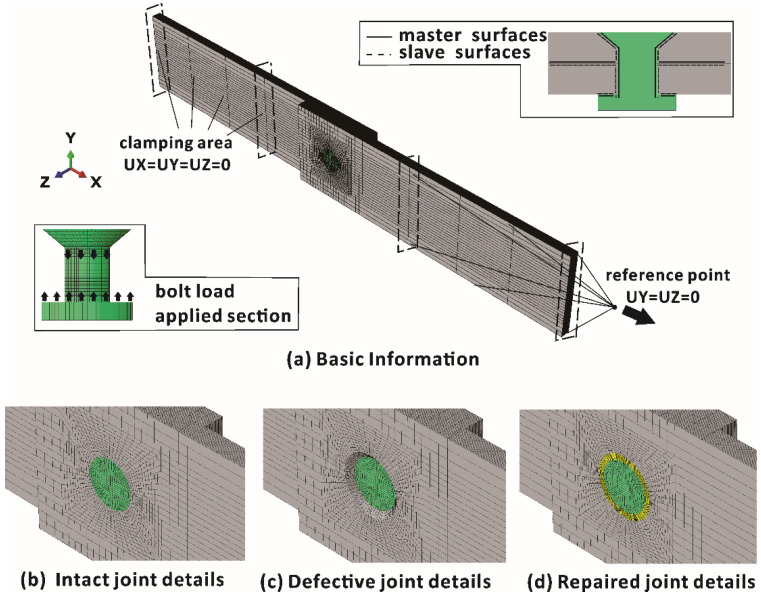
Finite element models: (**a**) basic information and details of (**b**) intact, (**c**) defective and (**d**) repaired samples.

To further analyze and explain the mechanical behaviors of the intact, defective and repaired samples, three corresponding FE models were created using Abaqus 6.13/Explicit, as shown in Figure 9. The models had the same geometries and initial mechanical properties as the specimens. Three-dimensional eight-node reduced integration solid elements (C3D8R) were used for model meshing. The hole edge mesh was refined to ensure calculation accuracy. After convergence verification, the minimum size of elements near the hole edges of laminates was set to 0.2 mm. In the thickness direction, each layer was represented by one solid element. Altogether 106,428 elements were used to discretize the joint. The clamping area of FE models was the same as that of experimental samples, which was shown in Figure 1. The boundary condition at the clamping area of the lower laminate was UX = UY = UZ = 0. To define the contacts, a surface-to-surface algorithm was implemented, which allowed pressure to be transferred between contacting surfaces and prevented contact areas from penetrating each other. The friction coefficient between all contacts was 0.2 [10,24]. The relationship between master surfaces and slave surfaces and the model details are shown in Figure 9.

The effect of bolt torque was simulated through the compression deformation of the bolt. According to the measurement of our colleagues, a torque of 1 Nm produced 76 micro-strains in the axial direction of the bolt. Therefore, during step 1 of the finite element model, a corresponding 0.0053 mm relative compression displacement was applied to the upper and lower sections of the bolt (see Figure 9). After step 1, an x-displacement was applied at the reference point of the clamping area of the upper laminate using a smooth step to achieve a quasi-static state during step 2.

The bolts and nuts in FE models were made of Ti-6Al-4V alloy (*E*_b_ = 108 GPa, *ν*_b_ = 0.33) and were considered as a single structure for simplification. The Johnson-Cook model was used to describe the plasticity of the bolt. The expression of the Johnson-Cook model is shown as follows [25]:

(1) $$\sigma =(A+B\varepsilon^{n})(1+C\ln{\dot{\varepsilon }}^{*})$$

where *σ* is the equivalent stress, *ε* is the equivalent plastic strain, ${\dot{\varepsilon }}^{*}$ is the equivalent strain rate and *A*, *B*, *C* and *n* are material parameters. The titanium alloy has values of *A* = 1098 MPa, *B* = 1092 MPa, *C* = 0.014 and *n* = 0.93 [26].

In the FE model for repaired samples, the filler material was tied with the composite laminates, assuming no significant debonding between the filler material and laminates (from digital camera observation during experiments and post-test failure analysis).

### 4.2. Failure Criteria and Degradation Rules

Hashin-type failure criteria [27] are widely used in fiber reinforced polymer material research. As shown in Table 4, Hashin-type failure criteria and constant degradation rules, based on a previous study [10], were employed in this study for T700/QY9611 composite materials via the Abaqus VUSDFLD subroutine.

Considering that the short fiber composite material for repair is isotropic, the maximum stress failure criteria and constant degradation rule were used for the filler material, as shown in Table 5.

Since the termination of the tests was caused by bolt fracture, damage of the bolt material was also considered in FE models. To this end, Johnson–Cook damage criteria in Abaqus/Explicit was used for predicting the damage initiation of Ti-6Al-4V alloy, where the general expression is shown as follows [28]:

(2) $${\bar{\varepsilon }}_{D}^{pl}=[d_{1}+d_{2}\exp(-d_{3}\eta )][1+d_{4}\ln(\frac{{\dot{\bar{\varepsilon }}}^{pl}}{{\dot{\bar{\varepsilon }}}_{0}})]$$

where ${\bar{\varepsilon }}_{D}^{pl}$ is the equivalent plastic strain at the onset of damage, *d*_1_–*d*_4_ are failure parameters, *η* is the stress triaxiality and ${\dot{\bar{\varepsilon }}}_{0}$ is the reference strain rate. The values of *d*_1_, *d*_2_, *d*_3_, *d*_4_ and ${\dot{\bar{\varepsilon }}}_{0}$ for the Ti-6Al-4V alloy are −0.09, 0.27, 0.48, 0.014 and 1, respectively [26]. After damage onset occurs, a fracture-energy-based criterion is applied during the damage evolution stage, as given by:

(3) $$d=1-\exp(-\int_{0}^{{\bar{u}}^{pl}}\frac{{\bar{\sigma }}_{y}{\dot{\bar{u}}}^{pl}}{G_{f}}dt)$$

where *d* is the damage variable and *G*_f_ is the fracture energy. Once the damage variable *d* of the element reaches 1, the element will be removed from the FE model.

## 5. Numerical Results and Discussions

### 5.1. Load–Displacement Curves

**Figure 10:**
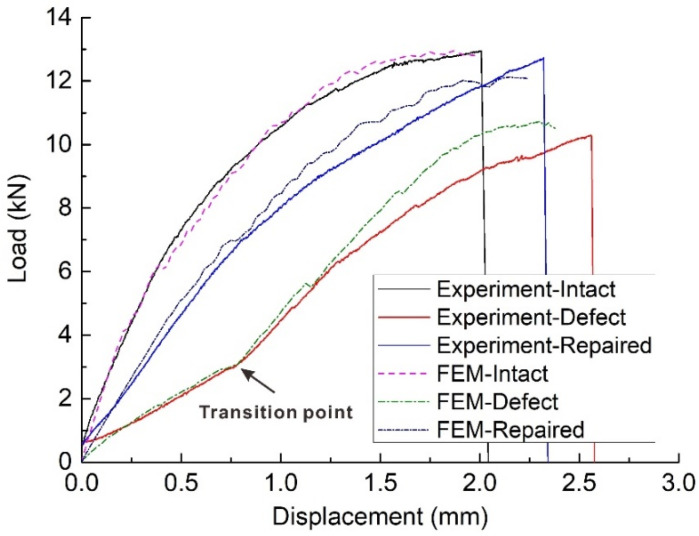
Comparison of the numerical and representative experimental load-displacement curves.

The obtained numerical results were compared with the experimental results to validate the FE models. The load–displacement curves are compared in Figure 10. When the bolt was completely broken, all calculations were stopped and the displacement–load curves reached their end points, which was the same as the experimental cases. The FE models accurately reproduced the non-linearity of the load–displacement responses caused by the degradation of mechanical properties. Additionally, the main feature of the experimental curve was also successfully captured. In the defective joints, the obvious transition points in the stiffness occurred at the same position (at the load of 3 kN) in both numerical and experimental curves.

In terms of stiffness and failure loads, a good agreement between the numerical and experimental data was also observed. The predicted failure loads of the intact, defective and repaired joints were 12.84, 10.50 and 12.08 kN, respectively, while the experimentally determined values were 12.94, 9.70 and 12.55 kN, respectively, which yielded the differences of −1%, 8% and −4%, respectively. The main reason for the differences between the predicted and experimental values may be that the bolt and nut were simplified into a whole structure for modelling to improve the computational efficiency. This simplification ignored the clearance and relative displacement between the bolt and nut. For defective joints, since the overall deformation was more severe, the effect of clearance and relative displacement on the predicted value was more significant. Therefore, compared with the intact and repaired joints, the calculation discrepancy of the defective joints was slightly larger.

### 5.2. Contact Behavior and Local Deformation

**Figure 11:**
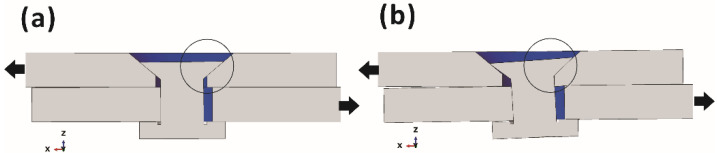
The contact behavior of a defective joint (**a**) before the transition point and (**b**) after the transition point.

The contact behavior was investigated in order to elucidate the reason for the transition point in the stiffness of defective joints, as shown in Figure 11. The over-tolerance defect resulted in large clearances and mismatched countersinks in the joints. Therefore, at a low applied load, the bolt shank did not come in contact with the inner surface of the hole. In this case, the load transfer of the upper laminate mainly depended on the contact with the countersink area, as shown in Figure 11a, which resulted in low joint stiffness. As the applied load increased, the clearance between the bolt shank and the upper laminate at the bearing side of the hole gradually decreased. When the bolt shank came into contact with the hole (see Figure 11b), the load could be transmitted through the bolt shank, leading to an increase in the stiffness of the entire joint. As a result, transition points could be observed in the load–displacement curves.

In the case of the repaired joints, the filler material compensated the large clearance and mismatched countersink, resulting in higher initial stiffness and no transition point in the load–displacement curve compared with the defective joints. However, since the stiffness of the filler material was lower than that of the laminate, stiffness of the repaired joints could not be completely restored to that of the intact joint.

**Figure 12:**
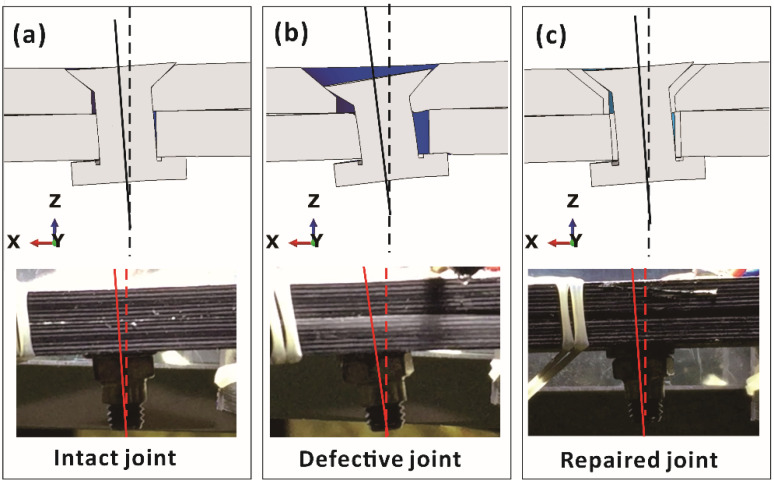
Numerical and experimental local deformations of bolts subjected to the same applied load (9 kN) for (**a**) intact, (**b**) defective and (**c**) repaired joints.

It should be noted that due to the large clearance and mismatched countersink in the defective joint, the bending deformation of the bolt in this joint was larger than those in intact and repaired joints under the same applied load, as shown in Figure 12. Large local deformations of the bolt tended to cause premature fracture, which resulted in a low failure load. The filler material in the repaired joint hindered the bending deformation and increased the failure load. The numerical results are consistent with the experimental results.

### 5.3. Failure Mechanisms

**Figure 13:**
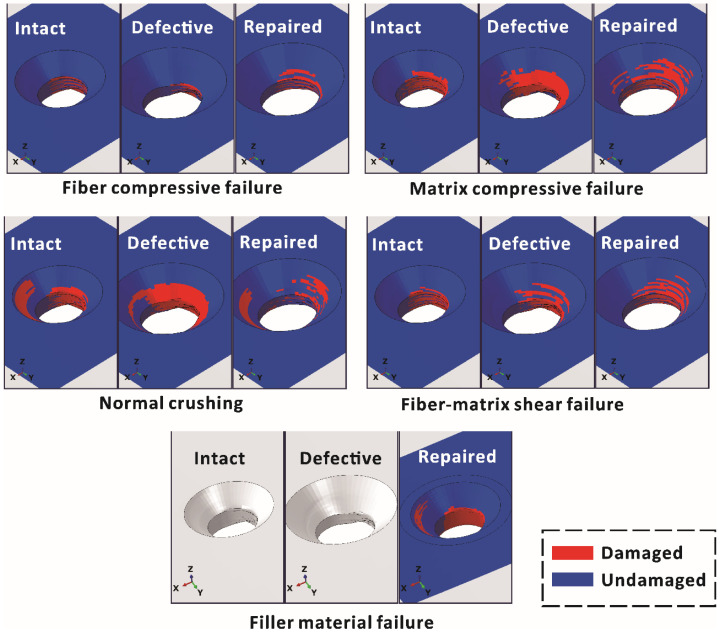
Simulated failure modes around the holes of upper laminates.

To analyze the failure of different joints, final damage distributions around the holes of upper laminates were simulated, as shown in Figure 13. For the sake of simplicity, only the main failure modes (fiber compressive failure, matrix compressive failure, normal crushing, fiber–matrix shear failure and filler material failure) are displayed.

The final damage was mainly located around the area between the cylindrical hole and the countersink, which was typical for bearing damage under unidirectional tensile loading. The predicted final damage location was similar to our experimental results and other FE studies [10,12], which validated our numerical models. Due to the secondary bending effect, the applied load was mainly transmitted to the cylindrical hole of the laminate via the bolt shank. Therefore, the location of fiber compressive failure in all three models was concentrated around the cylindrical hole, where the lower plies were damaged more seriously.

In the case of matrix compressive failure, the damage was also concentrated around the cylindrical hole due to the secondary bending effect. However, as the defective and repaired laminates (not including the short fiber composite material part) had mismatched countersinks, their countersinks were larger than that of the intact sample, while the depth of the cylindrical holes were shallower than that of the intact laminate. Therefore, compared with intact laminates, the countersink area beared a greater load and more damage in the defective and repaired laminates.

In the case of normal crushing, the distribution of material crushing was also dominated by secondary bending. The load exerted by the bolt shank caused damage mainly on the cylindrical hole, while the compressive load exerted by the bolt head damaged the top plies on the opposite side. For the same reason, the damage observed in defective and repaired laminates was located closer to the top ply than that in the intact laminates due to the mismatched countersink.

In the case of fiber–matrix shear failure, the final damage was mainly distributed around the cylindrical hole. However, in the case of filler material failure in the repaired laminate, the filler material compensated for the dimensional mismatch and improved the stress distribution, although the filler material around the cylindrical hole was eventually crushed, as observed in the experimental results.

## 6. Conclusions

In this study, a short fiber filling repair method was proposed to repair composite joints with defective holes. The mechanical performances of intact, defective and repaired joints under the tensile load were compared experimentally and numerically, and the results could be summarized as follows:

1. Hole edge defects would reduce the mechanical properties of the joints. Defective joints with over-tolerance countersink had a significantly lower failure load than the other samples (the average failure load of defective samples was only 74.9% of intact samples). Moreover, the initial stiffness of defective joints was lower than that of the other joints, where a transition point in the stiffness at about 3 kN was observed.
2. The average failure load of repaired samples was 96.9% of intact samples (the lower and upper limit of load capacity recovery ratio were 93.4% and 99.1%, respectively). The short fiber filling repair method proposed in this study could adequately restore the ultimate strength and partial stiffness of the samples, showing excellent repair efficiency.
3. Numerical analysis indicated that the low initial stiffness and transition point for defective joints were due to the load being transmitted by the bolt head instead of the bolt shank during initial deformation, which was caused by the large clearance and mismatched countersink. The short fiber material filled the geometric defect and improved the mechanical behavior of the repaired joints, resulting in a higher initial stiffness and failure load compared with the defective joints, which explained the repair mechanism.
4. Both the experimental and numerical results showed that the laminate failure of three types of joints mainly occurred around the hole and countersink; nevertheless, due to the effects of geometric defects and secondary bending, the failure distribution of defective joints differed slightly from the other joints.

## Figures and Tables

**Table 1:** Material properties of T700/QY9611.

Elastic Property	Value	Strength	Value
Longitudinal Young’s modulus *E*_11_ (GPa)	146	Longitudinal tensile strength *X*_T_ (MPa)	2391
Transverse Young’s modulus *E*_22_ = *E*_33_ (GPa)	104	Longitudinal compression strength *X*_C_ (MPa)	1410
Longitudinal shear modulus *G*_12_ = *G*_13_ (GPa)	6.45	Transverse tensile strength *Y*_T_ = *Z*_T_ (MPa)	67
Transverse shear modulus *G*_23_ (GPa)	3.37	Transverse compression strength *Y*_C_ = *Z*_C_ (MPa)	219
Longitudinal Poisson’s ratio *v*_12_ = *v*_13_	0.28	Longitudinal shear strength *S*_12_ = *S*_13_ (MPa)	94
Transverse Poisson’s ratio *v*_23_	0.30	Transverse shear strength *S*_23_ (MPa)	94
Density *ρ* (g/cm^3^)	1.6		

**Table 2:** Mechanical properties of the short fiber composite material.

Mechanical Property	Value
Elastic modulus *E*_sf_ (GPa)	10
Poisson’s ratio *v*_sf_	0.36
Compression strength *σ*_sf_ (MPa)	290
Density *ρ*_sf_ (g/cm^3^)	1.2

**Table 3:** Failure loads of the intact, defective and repaired composite joints.

Sample Types	Failure Load (kN)	Average Failure Load (kN)	Standard Deviation (kN)	Variation Coefficient (%)
Intact samples	12.95	12.94	0.60	4.6
	13.54			
	12.34			
Defective samples	9.52	9.70	0.52	5.4
	10.29			
	9.30			
Repaired samples	12.08	12.55	0.41	3.2
	12.82			
	12.74			

**Table 4:** Failure criteria and degradation rules for T700/QY9611 composite materials.

Failure Mode	Failure Criterion	Degradation Rule
Fiber tensile failure (FT)	$f_{FT}={\left(\frac{\sigma_{11}}{X_{T}}\right)}^{2}+{\left(\frac{\tau_{12}}{S_{12}}\right)}^{2}+{\left(\frac{\tau_{13}}{S_{13}}\right)}^{2}\geq 1,\;(\sigma_{11}>0)\;$	*E*_11_ = 0.1*E*_11_, *v*_12_ = 0.1*v*_12_, *v*_13_ = 0.1*v*_13_, *G*_12_ = 0.1*G*_12_, *G*_13_ = 0.1*G*_13_
Fiber compressive failure (FC)	$f_{FC}={\left(\frac{\sigma_{11}}{X_{C}}\right)}^{2}\geq 1,\;(\sigma_{11}<0)\;$	
Matrix tensile failure (MT)	$f_{MT}={\left(\frac{\sigma_{22}}{Y_{T}}\right)}^{2}+{\left(\frac{\tau_{12}}{S_{12}}\right)}^{2}+{\left(\frac{\tau_{23}}{S_{23}}\right)}^{2}\geq 1,\;(\sigma_{22}>0)\;$	*E*_22_ = 0.3*E*_22_, *v*_12_ = 0.3*v*_12_, *v*_23_ = 0.3*v*_23_, *G*_12_ = 0.3*G*_12_, *G*_23_ = 0.3*G*_13_
Matrix compressive failure (MC)	$f_{MC}={\left(\frac{\sigma_{22}}{Y_{C}}\right)}^{2}+{\left(\frac{\tau_{12}}{S_{12}}\right)}^{2}+{\left(\frac{\tau_{23}}{S_{23}}\right)}^{2}\geq 1,\;(\sigma_{22}<0)\;$	
Delamination in tension (DT)	$f_{DT}={\left(\frac{\sigma_{33}}{Z_{T}}\right)}^{2}+{\left(\frac{\tau_{13}}{S_{13}}\right)}^{2}+{\left(\frac{\tau_{23}}{S_{23}}\right)}^{2}\geq 1,\;(\sigma_{33}>0)\;$	*E*_33_ = 0.2*E*_33_, *v*_23_ = 0.2*v*_23_, *v*_13_ = 0.2*v*_13_, *G*_13_ = 0.2*G*_13_, *G*_23_ = 0.2*G*_23_
Normal crushing (NC)	$f_{NC}={\left(\frac{\sigma_{33}}{Z_{C}}\right)}^{2}+{\left(\frac{\tau_{13}}{S_{13}}\right)}^{2}+{\left(\frac{\tau_{23}}{S_{23}}\right)}^{2}\geq 1,\;(\sigma_{33}<0)\;$	
Fiber-matrix shear failure (SS)	$f_{SS}={\left(\frac{\sigma_{11}}{X_{C}}\right)}^{2}+{\left(\frac{\tau_{12}}{S_{12}}\right)}^{2}+{\left(\frac{\tau_{13}}{S_{13}}\right)}^{2}\geq 1,\;(\sigma_{11}<0)\;$	*v*_12_ = 0.2*v*_12_, *G*_12_ = 0.2*G*_12_

**Table 5:** Failure criteria and degradation rules for the short fiber composite material.

Failure Criterion	Degradation Rule
$f=\left|\sigma_{11}\right|\geq \sigma_{\mathrm{sf}}$ $f=\left|\sigma_{22}\right|\geq \sigma_{\mathrm{sf}}$ $f=\left|\sigma_{33}\right|\geq \sigma_{\mathrm{sf}}$	*E*_sf_ = 0.4*E*_sf_

## Data Availability

Not applicable.
